# Differentiation of NaCl, NaOH, and β-Phenylethylamine Using Ultraviolet Spectroscopy and Improved Adaptive Artificial Bee Colony Combined with BP-ANN Algorithm

**DOI:** 10.1021/acsomega.3c00271

**Published:** 2023-03-20

**Authors:** Angxin Tong, Xiaojun Tang, Haibin Liu, Honghu Gao, Xiaofei Kou, Qiang Zhang

**Affiliations:** † School of Management Engineering, Zhengzhou University of Aeronautics, Zhengzhou 450046, China; ‡ School of Electrical Engineering, Xi’an Jiaotong University, Xi’an 710049, China; § Delixi Group Co., Ltd., Wenzhou 325604, China

## Abstract

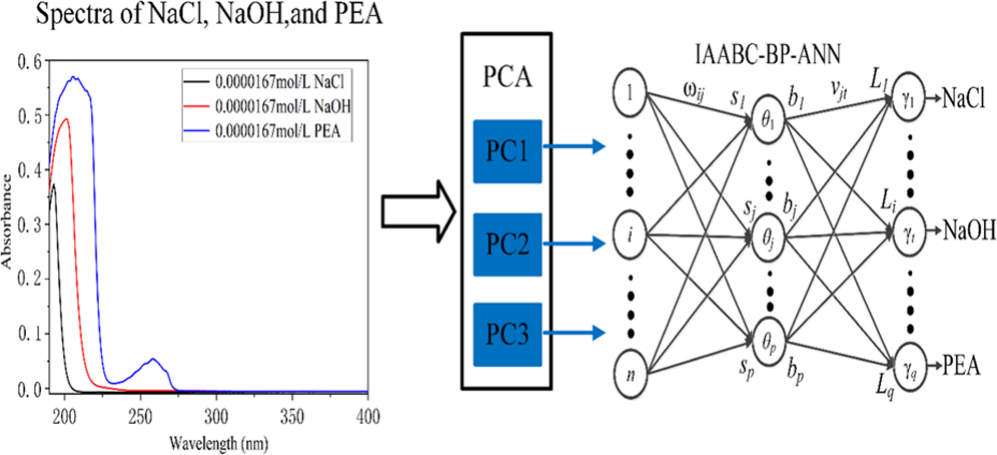

The aim of this study is to enhance the classification performance of the back-propagation-artificial neural network (BP-ANN) algorithm for NaCl, NaOH, β-phenylethylamine (PEA), and their mixture, as well as to avoid the defects of the artificial bee colony (ABC) algorithm such as prematurity and local optimization. In this paper, a method that combined an improved adaptive artificial bee colony (IAABC) algorithm and BP-ANN algorithm was proposed. This method improved the ABC algorithm by adding an adaptive local search factor and mutation factor; meanwhile, it can enhance the abilities of the global optimization and local search of the ABC algorithm and avoid prematurity. The extracted score vectors of the principal component of the ultraviolet (UV) spectrum were used as the input variable of the BP-ANN algorithm. The IAABC algorithm was used to optimize the weight and threshold of the BP-ANN algorithm, and the iterative algorithm was repeated until the output accuracy was reached. The output variable was the classification results of NaCl, NaOH, PEA, and the mixture. Meanwhile, the proposed IAABC-BP-ANN algorithm was compared with discriminant analysis (DA), sigmaid-support vector machine (SVM), radial basis function-SVM (RBF-SVM), BP-ANN, and ABC-BP-ANN. Then, the above algorithms were used to classify NaCl, NaOH, PEA, and the mixture, respectively. In the experiment, four indicators, accuracy, recall, precision, and F-score, were used as the evaluation criteria. In addition, the regression equation parameters of the mixture for the testing set were obtained by BP-ANN, ABC-BP-ANN, and IAABC-BP-ANN models. All of the results showed that IAABC-BP-ANN exhibits better performance than other algorithms. Therefore, IAABC-BP-ANN combined with UV spectroscopy is a potential identification tool for the detection of NaCl, NaOH, PEA, and the mixture.

## 1 Introduction

β-Phenylethylamine (PEA) is an important organic synthesis intermediate. Its derivatives are widely used in the fields of dyes, medicine, emulsifiers, and spices.^1^ During the synthetic processing of PEA, NaOH is used as a reactant to synthesize PEA, and the final product usually contains PEA, NaCl, and NaOH.^2^ At present, the main detection methods of PEA include high-performance liquid chromatography, gas chromatography, capillary electrophoresis, ion chromatography–mass spectrometry, thin-layer chromatography, and so on.^3^ However, the above methods are time-consuming and cumbersome and have high requirements for the operation level of experimental personnel, detection environment, and chromatographic plate, which can not meet the requirements of simplicity, rapidity, and on-site. In contrast, ultraviolet (UV) spectroscopy has the advantages of fast real-time detection, no chemical reagent, low cost, no secondary pollution, and online in situ measurement.^3^ In addition, scholars at home and abroad rarely identify the substance types of the final PEA products. Therefore, the identification of NaCl, NaOH, PEA, and their mixtures is of great significance for the synthesis and qualitative measurement of PEA.

For the identification of substance types, the common method is to take the chemical composition of substance samples as the input data and then establish the differentiated characteristics of products for classification in combination with pattern recognition technology, which is of great significance for identifying the types, geographical sources, and authenticity of products.^4^ The main disadvantages of chemical methods are expensive equipment; usually involving large operation or maintenance costs; and using a variety of reagents for the extraction of organic compounds, the mineralization of samples, or the analysis of derivatives.^4^

Given this, for the identification of substance types, scholars at home and abroad applied new detection methods such as infrared spectroscopy,^5−8^ fluorescence spectroscopy,^9^ hyperspectral imaging technology,^10,11^ terahertz spectroscopy,^12−15^ laser-induced breakdown spectroscopy^16,17^ and Raman spectroscopy^18,19^ to the classification of industrial and agricultural products. Meanwhile, domestic and foreign scholars combined the above detection methods with supervised pattern recognition technologies such as discriminant analysis (DA), support vector machine (SVM), and artificial neural network (ANN) to successfully distinguish human faces,^20^ voice signal,^21^ varieties of biomedical,^22,23^ species of farm crops,^24,25^ types of food,^26^ and species of oil products.^27,28^ Although these methods combined with pattern recognition technology successfully classified and identified different substances, the above detection technology still has some disadvantages, such as a long measurement cycle, slow measurement speed, sample pretreatment, secondary pollution, and so on.^29^ In this context, UV spectroscopy combined with supervised pattern recognition technologies such as DA, SVM, and ANN was successfully used to identify varieties of different wines,^30,31^ the origin of pepper,^32^ the shape of anemone,^33^ and species of tea.^4^ Tong et al. successfully identified NaCl, NaOH, PEA, and their mixtures by using the artificial bee colony-back-propagation-ANN (ABC-BP-ANN) algorithm with UV spectroscopy.^34^

In recent years, ANN combined with hybrid machine learning algorithms have been widely used in different fields, such as the heating load of buildings’ energy efficiency,^35^ prediction of oil recovery,^36^ permeability and porosity of petroleum reservoirs,^37−39^ thermal conductivity ratio and dynamic viscosity of alumina/water nanofluid,^40,41^ dissolved calcium carbonate concentration in oil field brines,^42^ permeability impairment due to scale deposition,^43^ efficiency of chemical flooding in oil reservoir,^44^ and oil well production performance.^45^

In addition, some scholars have used unsupervised *t*-SNE machine learning algorithms to classify large amounts of data. Raza et al. presented an unsupervised *t*-SNE machine learning algorithm that can automatically classify and rationalize chemical trends in PFAS structures.^46^ Halladin-Dabrowska et al. successfully used visual analysis of *t*-SNE-based plots to identify the subset of reference database having specific visual artifacts and patterns, correlated with a significant probability of containing errors.^47^

However, DA can not continue to be used when the centers of various categories overlapped; SVM is difficult to solve the problem of multiclassification; ANN is easy to fall into local minimum and slow convergence; and artificial bee colony (ABC) is prone to fall into the prematurity and local optimization.

Therefore, in this paper, we proposed a new pattern recognition technology based on the comparison of supervised pattern recognition technologies such as DA, sigmoid SVM, radial basis function-SVM (RBF-SVM), BP-ANN, and ABC-BP-ANN,^48,49^ which used improved adaptive artificial bee colony (IAABC) to optimize the weight and threshold of BP-ANN. From the obtained results of four indicators, accuracy, recall, precision, and F-score, it can be seen that UV spectroscopy combined with IAABC-BP-ANN is a simple, rapid, and reliable classification method for distinguishing NaCl, NaOH, PEA, and their mixtures. The research results provide a new theoretical basis for the synthesis and qualitative measurement of PEA.

## 2 Experiment and Method

### 2.1 Sample Preparation and Testing

NaCl, NaOH, and PEA of analytical grade used in the experiment were purchased from Shanghai Aladdin Biochemical Technology Company. First, 1 mol/L NaCl, 2 mol/L NaOH, and 0.0312 mol/L PEA standard solutions were prepared, respectively, and 30 samples for each type with different concentrations were obtained by diluting standard solutions with deionized water. The resulting concentration ranges of NaCl, NaOH, and PEA were 0.00000352–0.5, 0.00000762–0.5, and 0.00000306–0.00891 mol/L, respectively. Finally, seven mixtures with different mole fractions (m/m) were prepared (the mole fraction of the mixture refers to the percentage of PEA in the total substance of the mixture), and the concentration ranged from 0 to 60% m/m. 30 sample solutions were prepared at different concentrations, and the corresponding spectral data were obtained, which contained spectral data of 210 groups. All samples were prepared at room temperature, which was approx. 20 °C.

The UV spectra of NaCl, NaOH, PEA, and the mixture samples were determined by a UV-2900 spectrophotometer purchased from Shanghai Sunny Hengping Scientific Instrument Co., Ltd., and the deionized water was used as the reference sample. UV-2900 has two identical quartz cuvettes with a 10 mm optical path. The measurement range of the spectrum was 190–400 nm, the sampling interval was 1 nm, and the scan rate was 1 nm/s. Each sample was scanned 5 times to calculate the average spectrum.

### 2.2 Data Analysis

20 samples for each type with different concentrations were used as the training set, and the other 10 samples were used as the testing set. Principal component analysis (PCA) was used to reduce the number of variables that needed to be used in the classification model. DA, sigmoid SVM, RBF-SVM, BP-ANN, ABC-BP-ANN, and IAABC-BP-ANN were used for classification. All programs were run on MATLAB R2021a (American MathWorks Co., Ltd.) and Statistica 8.0 software package (American STAT SOFT Co., Ltd.) was used for statistical analysis.

### 2.3 Basic Artificial Bee Colony Algorithm

The standard ABC algorithm consists of employed bees, onlooker bees, and scout bees. The ultimate goal of the ABC algorithm is to find the most abundant honey source. Assuming that all problems in the ABC algorithm are solved under the condition of *D*-dimension vector space, and the total number of honey sources is *N*. The initial position of the honey source is shown in eq 1
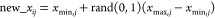
1where *i* and *j* are randomly generated, *i* = (1,2,···,*N*), *j* = (1,2,···,*D*), and *i* ≠ *j*; rand(0,1) is a number randomly generated in the range (0,1); new_*x*_*ij*_ is the position of the initial solution; and *x*_max,*j*_ and *x*_min,*j*_ are the upper and lower bounds of the *j*-dimension, respectively.

Assuming that a new honey source of high quality is found, the probability of the honey source being selected is shown in eq 2
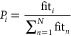
2where fit_*i*_ represents the fitness value corresponding to the *i*th honey source, and it is shown in eq 3
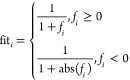
3If the quality of the honey source has not been improved after several circulations, in order to find a new position of the honey source from the old honey source, the employed bees will be transformed into scout bees. According to eq 4, the new position of the honey source is searched.
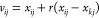
4where *k* and *j* are randomly generated, *k* = (1,2,···,*N*), *j* = (1,2,···,*D*), and *k* ≠ *i*; *r* is a number randomly generated in the range [−1,1]; ν_*i*_ is the new neighboring honey source to *x*_*i*_; and *x*_*ij*_ and *x*_*kj*_ are the positions of the referenced honey source *x*_*i*_ and randomly selected honey source *x*_*k*_ in dimension *j*, respectively.

### 2.4 Improved Adaptive Artificial Bee Colony (IAABC) Algorithm

In this paper, the adaptive local search factor and mutation factor are added to improve the ABC algorithm to enhance the global optimization ability and local search ability of the algorithm, so as to avoid prematurity.

#### 2.4.1 Adaptive Search Factor

To avoid falling into the local optimization of the ABC algorithm, the adaptive local search factor ω is introduced in the initial search stage of the ABC algorithm. And the local search is enhanced by adaptively adjusting the population update step size to balance the global and local search abilities of the ABC algorithm. The specific method is to update eqs 4–5.
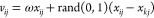
5

The main purpose of introducing ω is to avoid prematurity and improve the convergence speed of the ABC algorithm. The change of ω is shown in eq 6.
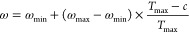
6where ω_min_ and ω_max_ represent the minimum and maximum values of inertia weight, respectively; *c* is the current number of iterations; and *T*_max_ is the maximum number of iterations.

#### 2.4.2 Mutation Factor

To improve the ability of global optimization and accuracy of the ABC algorithm, the Levy mutation factor is introduced into the ABC algorithm. Compared with other operators such as the Gaussian mutation operator, the Levy mutation factor greatly enhances the global optimization ability of the ABC algorithm and avoids prematurity. The introduction of the Levy mutation factor enhances the global optimization ability of the ABC algorithm based on the adaptive search factor. The specific method is to add the Levy mutation operator to eq 5 and update it to eq 7
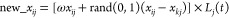
7where *L*_*j*_(*t*) is a random number subject to Levy distribution.

To verify the performance of the IAABC algorithm in this paper, the Griewank function is used for testing function, and the function expression was shown as follows
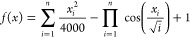
8where *x*_*i*_ ∈ [−600,600], and there is a globally optimal solution *f*(**0**) = 0.

**Figure 1:**
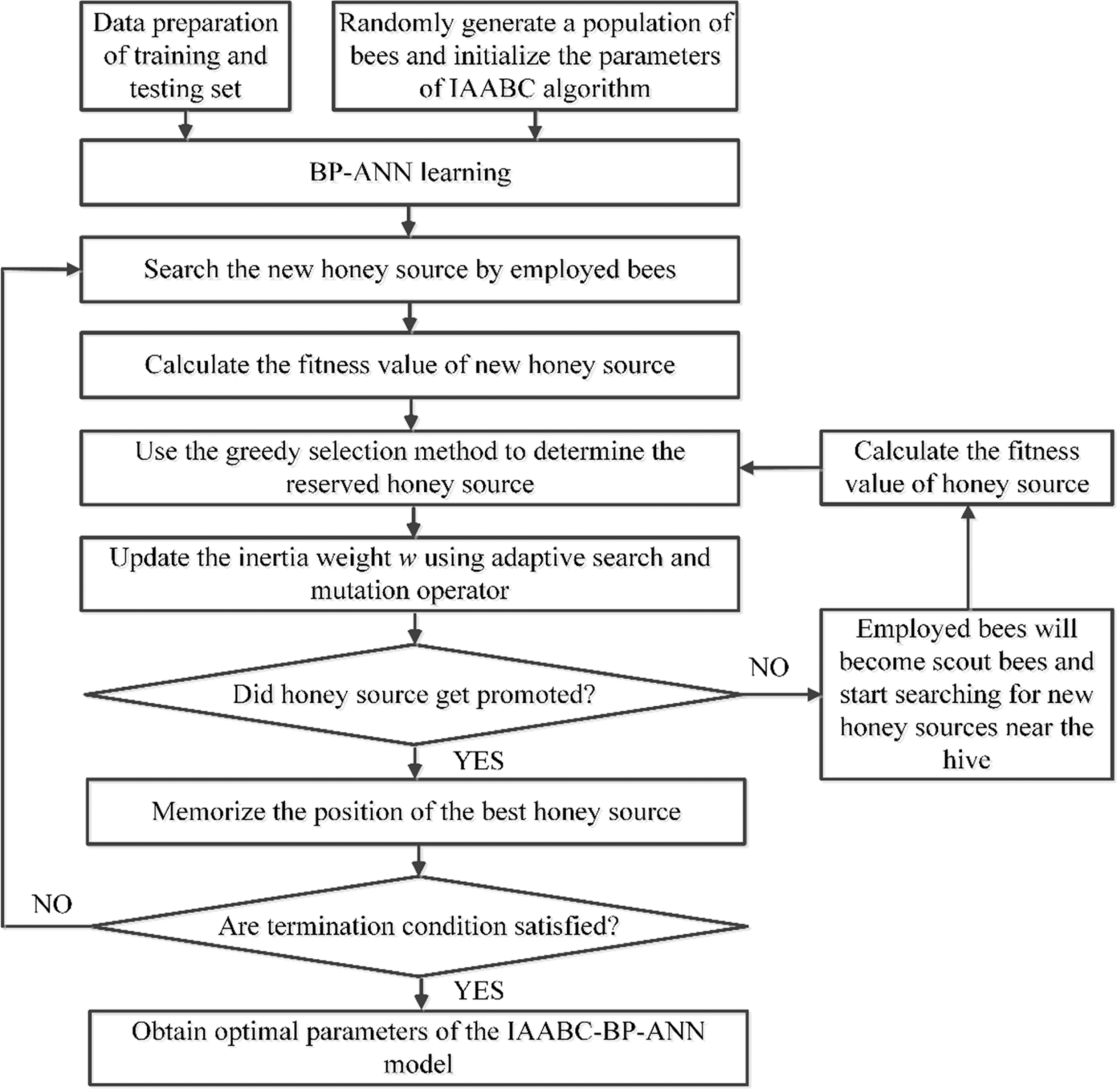
Process of optimizing the BP-ANN model with the IAABC algorithm.

The process of optimizing the BP-ANN model with the IAABC algorithm is presented in Figure 1, which can be described as follows:

Step 1: Data preparation of training and testing set.

Step 2: Initialize the parameters of the IAABC algorithm, which mainly include the number of honey sources *N*, the maximum number of iterations MaxCycle, and the maximum number of retention limit. The error limitation of fitness function and the restricted range of inertia weight ω.

Step 3: BP-ANN learning. The main components of the UV spectra of NaCl, NaOH, and PEA were used as inputs to train the BP-ANN network.

Step 4: The new honey source was searched by employed bees according to Formula 7, then the fitness value of new honey source v was calculated and the honey source was updated.

Step 5: The greedy selection method was used to determine the reserved honey source according to the fitness value of the new honey source v.

Step 6: Adopt adaptive search and mutation operator. The inertia weight ω was updated generation by generation by Formula 6, and the Levy mutation operator was a random number subject to the Levy distribution.

Step 7: Compare the fitness values of the new honey source vi and the honey source xi, and replace them if the fitness value of the new honey source vi was better than hone source xi.

Step 8: If the honey source xi did not improve, check whether there was an abandoned honey source and then replace the abandoned honey source by generating a random honey source by Formula 1. Employed bees will become scout bees and start searching for new honey sources near the hive; meanwhile, the position of the honey source was updated by Formula 7.

Step 9: Record the fitness value of each honey source and obtain the best honey source.

Step 10: Check whether the termination condition of the cycle was satisfied, that is, whether the maximum number of cycle times Maxcycle and the specified precision were reached. Otherwise, return to Step 2 to continue.

### 2.5 Forecasting Model of Substance Species Based on IAABC-BP-ANN Algorithm

**Figure 2:**
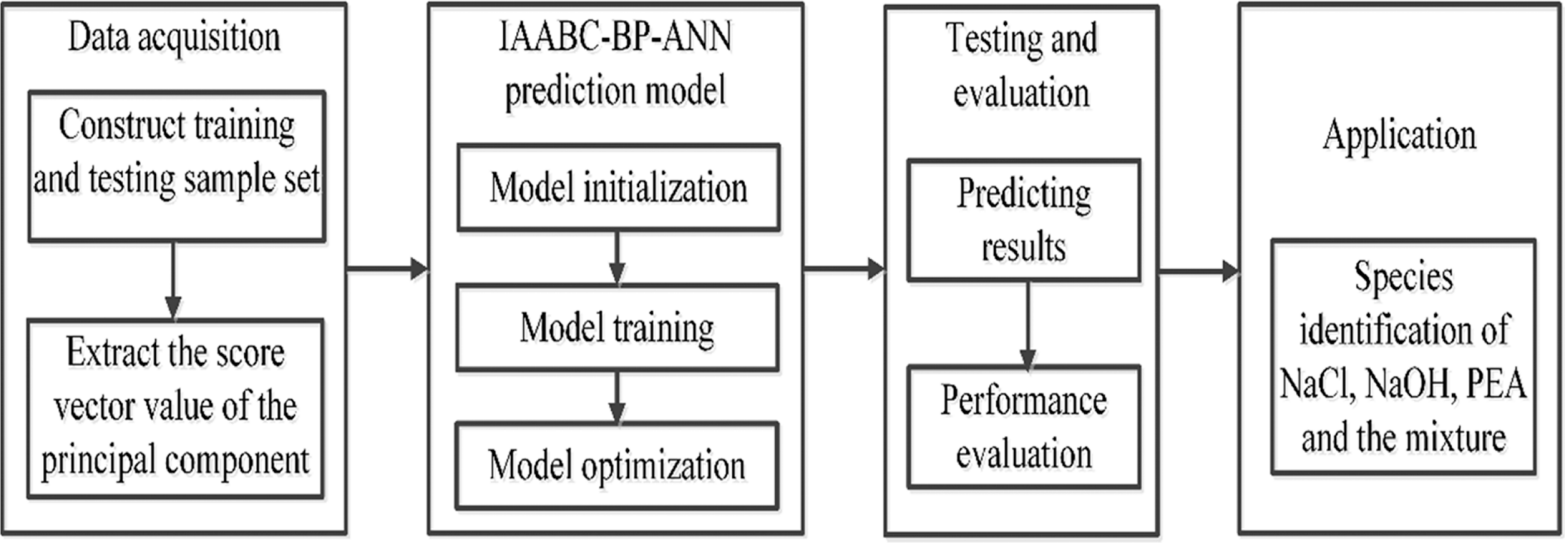
Implementation process of predicting species of NaCl, NaOH, PEA, and the mixture.

Based on the above algorithm, this paper uses the IAABC-BP-ANN algorithm to discriminate the species of NaCl, NaOH, PEA, and the mixture. The improved algorithm has the advantages of generalization mapping and global iterative search ability. The implementation process of predicting species of NaCl, NaOH, PEA, and the mixture based on the IAABC-BP-ANN algorithm is shown in Figure 2. The prediction system includes data acquisition, establishment and optimization of the prediction model, testing and evaluation, and application.

The implementation process based on the IAABC-BP-ANN algorithm model can be described as follows:

Step 1: Construct a training sample and a testing sample set. Meanwhile, extract the score vector value of the principal component.

Step 2: Initialize the parameters of the IAABC algorithm and set the number of honey sources *N*, the maximum number of iterations MaxCycle, the restricted range of inertia weight ω, etc.

Step 3: Train BP-ANN on the training sample set, obtain the parameters of the BP-ANN algorithm by IAABC, and obtain the prediction model based on the IAABC- BP-ANN algorithm.

Step 4: Utilize the testing sample set to test and evaluate the performance of the prediction model.

Step 5: Adopt the IAABC-BP-ANN algorithm to realize species identification of NaCl, NaOH, PEA, and the mixture.

### 2.6 Evaluation Criteria

To evaluate the proposed method more comprehensively, confusion matrix, accuracy, recall, precision, and F-score were used.

**Figure 3:**
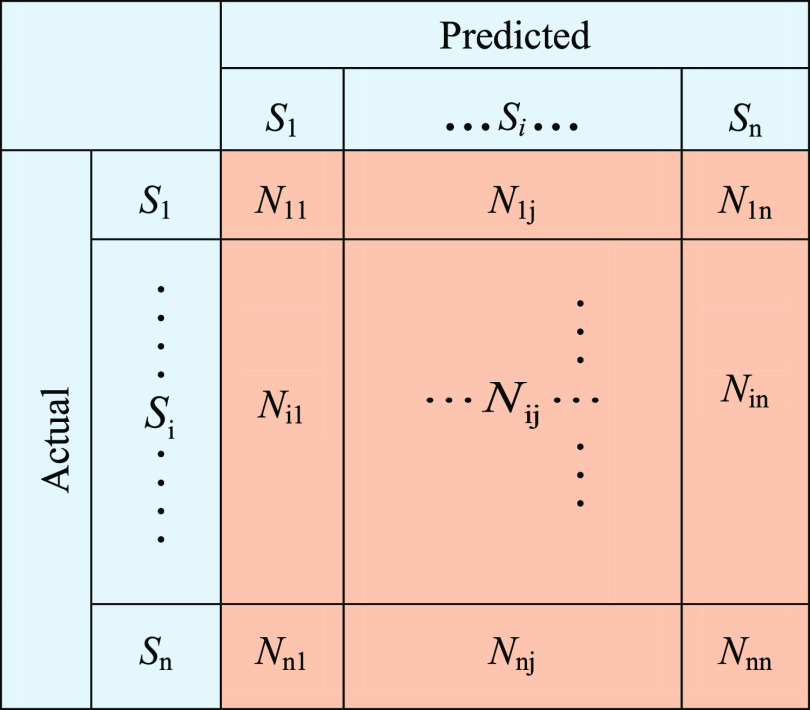
Confusion matrix.

The diagram of a confusion matrix predicted classification is shown in Figure 3. In the confusion matrix, each column of the matrix represents the predicted value of the sample and each row represents the actual value of the sample. *N*_*ij*_ represents the number of samples that actually belong to class *i* but are misclassified as class *j*. *N*_*jj*_ represents the number of samples of class *j* that are correctly classified. Therefore, according to the diagram of the confusion matrix predicted classification, it can be intuitively judged that the more samples are on the diagonal, the better the recognition effect will be.

From the confusion matrix, four evaluation indicators, accuracy, recall, precision, and F-score, were obtained. They can be defined as follows
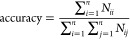
9
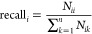
10
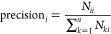
11
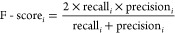
12Accuracy refers to the percentage of the total sample that is correctly predicted. Recall is an indicator for the original sample, which indicates how many positive examples in the original sample are predicted correctly. Precision is an indicator for the predicted results, which indicates how many samples of the predicted positive class are correct. F-Score is defined as the harmonic average of accuracy and recall. The value of the F-Score ranges from 0 to 1, where 1 represents the best output of the model and 0 represents the worst output of the model. A good classification model should have all high accuracy, recall, precision, and F-Socre.

To compare the predictive classification ability of the constructed models, the root mean square error of prediction (RMSEP), relative error of prediction (REP), and correlation coefficient (*R*^2^) were obtained. They can be defined as follows
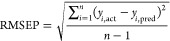
13
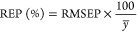
14
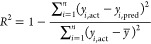
15where *n* is the number of listed samples, *y*_*i*,act_ is the actual value of the *i*th sample, *y*_*i*,pred_ is the predicted value of the *i*th sample in the model, and *y̅* represents the average value of all of the samples.

## 3 Results and Discussion

### 3.1 UV Absorption Spectra of Single Components and Their Mixtures

**Figure 4:**
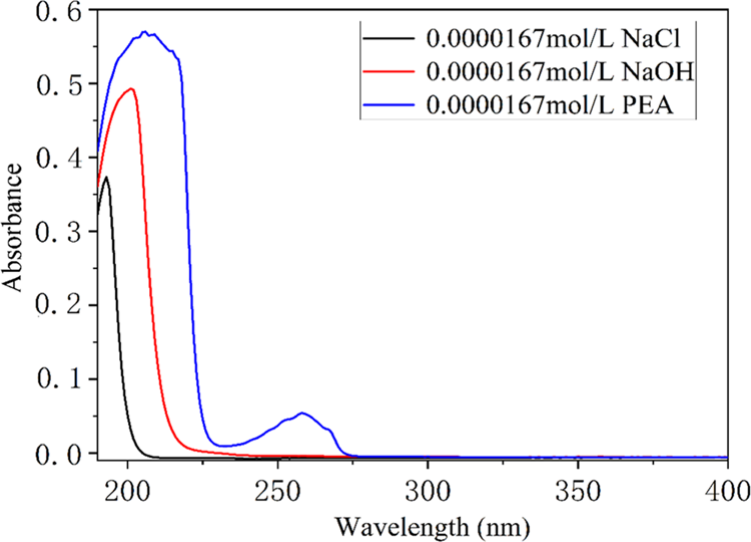
UV absorption spectra of NaCl, NaOH, and PEA at 0.0000167 mol / L.

Figure 4 shows the UV absorption spectra of NaCl, NaOH, and PEA with the same concentration, which was 0.0000167 mol/L. As can be seen from Figure 4, in the wavelength range of 190–400 nm, the characteristic absorption peaks of PEA were at 210 and 258 nm, respectively. The characteristic absorption peak of NaOH was at 202 nm. The characteristic absorption peak of NaCl was at 197 nm. The absorption peak of PEA was the strongest, followed by NaOH, and the absorption peak of NaCl was the weakest. Different substances contained different characteristic absorption peaks, and these characteristic absorption peaks can be used as the “fingerprint” of substances to identify the species of substances. Therefore, NaCl, NaOH, and PEA can be distinguished intuitively according to their different characteristic absorption peaks. However, the characteristic absorption peaks of NaCl and NaOH are very similar, and the characteristic absorption peaks of NaCl and NaOH solutions for different concentrations often overlapped with each other. At this time, the recognition of NaCl and NaOH by using the characteristic absorption peak may lead to misjudgment.

**Figure 5:**
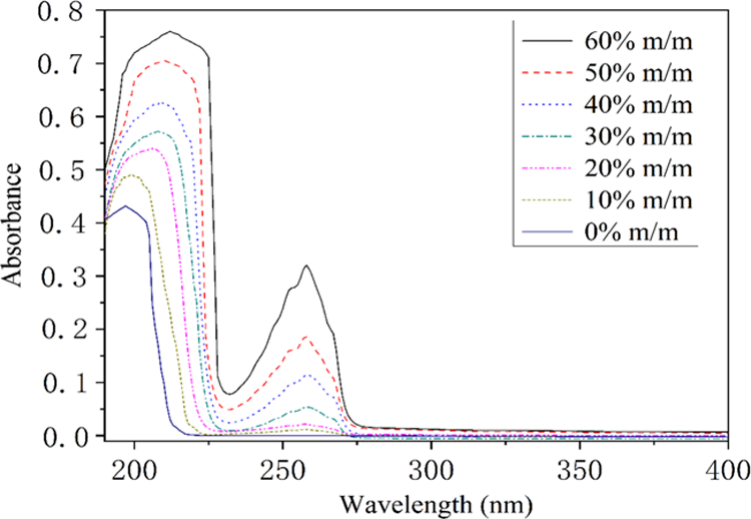
UV absorption spectra of the mixtures at seven different concentrations.

Figure 5 shows the UV absorption spectra of seven mixtures at different concentrations. As can be seen from Figure 5, the characteristic absorption peaks of the mixture were at 210 and 258 nm, respectively, when the concentration of PEA was higher, which was consistent with the characteristic absorption peaks of PEA. Therefore, the characteristic absorption peak of the mixture can be used to determine whether it contained PEA when the content of PEA in the mixture is higher. In addition, the intensity of the absorption peak decreased with the decrease of the concentration of PEA in the mixture, and the characteristic absorption peak of the mixture disappeared at 258 nm when the concentration of PEA in the mixture was less than 10% m/m. Therefore, it is impossible to judge whether the mixture contained PEA when the concentration of PEA in the mixture was less than 10% m/m.

According to the above analysis, it can be found that there are some limitations to distinguishing them only by relying on the absorption peak of the substance when the absorption peak of the measured substance was very similar and the concentration of the measured substance decreased. It is necessary to distinguish them by pattern recognition technology, which can effectively overcome the limitations of identifying NaCl, NaOH, PEA, and their mixtures by the absorption peak.

### 3.2 Extraction of the Score Vector of the Principal Component

**Figure 6:**
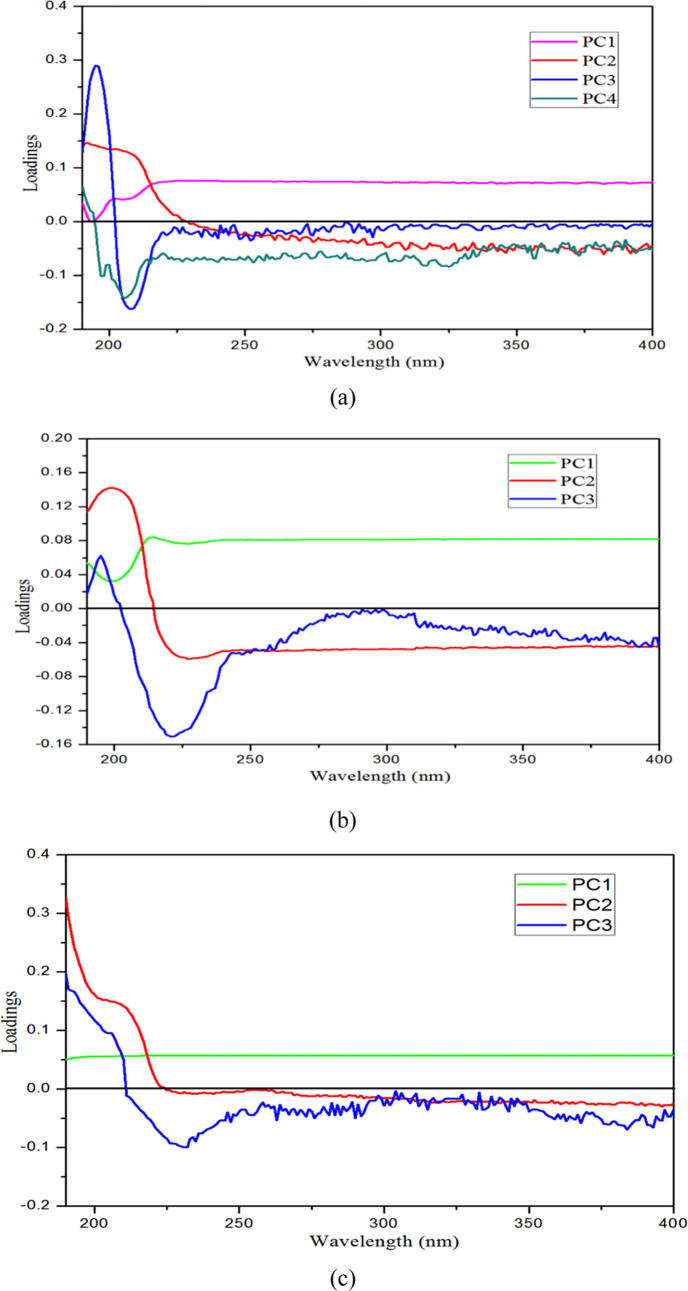
Loading vectors of the principal component (PC) of NaCl, NaOH, and PEA. (a) Loading vectors of NaCl in the four first principal components (PCs). (b) Loading vectors of NaOH in the three first PCs. (c) Loading vectors of PEA in the three first PCs.

Principal component analysis (PCA) is used to obtain linear combinations of the original variables called principal components (PCs). Meanwhile, the loading vectors and score vectors are also obtained by PCA. Figure 6 shows the loading vectors of the extracted principal components of NaCl, NaOH, and PEA. As can be seen from Figure 6a, the absorbances in the ranges 190–400, 190–230, 190–202, and 190–195 nm are highly correlated with the four first principal components of NaCl, respectively. The variance contribution rates of the four first principal components are 63.83, 25.51, 7.30, and 1.33%, respectively, and the cumulative contribution rate of the three first principal components is 96.64%. As can be seen from Figure 6b, the absorbances in the ranges 190–400, 190–220, and 190–205 nm are highly correlated with the three first principal components of NaOH, respectively. The variance contribution rates of the three first principal components are 74.75, 24.38, and 0.31%, respectively, and the cumulative contribution rate of the three first principal components is 99.44%. As can be seen from Figure 6c, the absorbances in the ranges 190–400, 190–225, and 190–210 nm are highly correlated with the three first principal components of PEA, respectively. The variance contribution rates of the three first principal components are 87.88, 10.77, and 1.30%, respectively, and the cumulative contribution rate of the three first principal components is 99.95%. It can be seen that the three first principal components of NaCl, NaOH, and PEA contain most of the spectral information.

**Figure 7:**
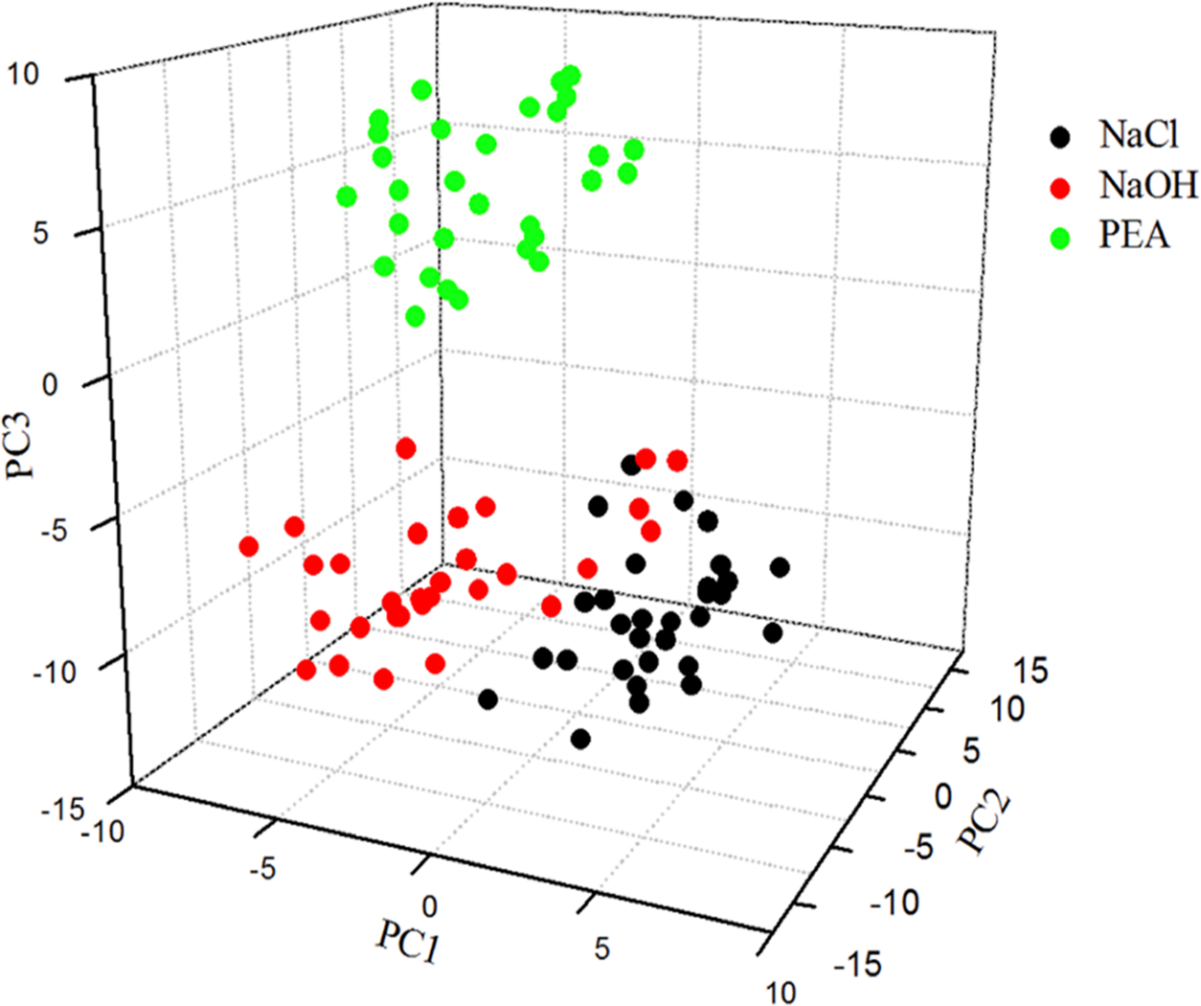
Distribution of score vector values in the space of the three first PCs.

Figure 7 shows the distribution of the score vector values of NaCl, NaOH, and PEA in the space formed by the three first principal components. As can be seen from Figure 7, the score vectors of the first and third principal components of PEA are negative and positive, respectively. Therefore, the species of PEA, NaCl, and NaOH can be distinguished according to the score vectors of the first and third principal components. The score vectors of the three first principal components of NaCl and NaOH overlapped partially, and the species of NaCl and NaOH can be distinguished according to the first and second score vector values. Given these trends, a supervised pattern recognition method can be used to classify the species of NaCl, NaOH, PEA, and their mixtures. The score vectors of the three first principal components are used as the input variables, and the species of samples are used as the output variables.

### 3.3 Classification Model of Supervised Pattern Recognition

The proposed IAABC algorithm in this paper is implemented with Matlab 2021a programming language. The optimal IAABC parameters after adjustment are as follows: the number of bees *N* is 35, the dimension of honey source *D* is 5, the maximum number of iterations Maxcycle is 2000, the maximum number of retention limit is 100, the inertia weights ω_max_ = 1.05 and ω_min_ = 0.15, and the error precision of the fitness function is 0.000001. The IAABC algorithm is run 30 times independently to eliminate the randomness.

**Figure 8:**
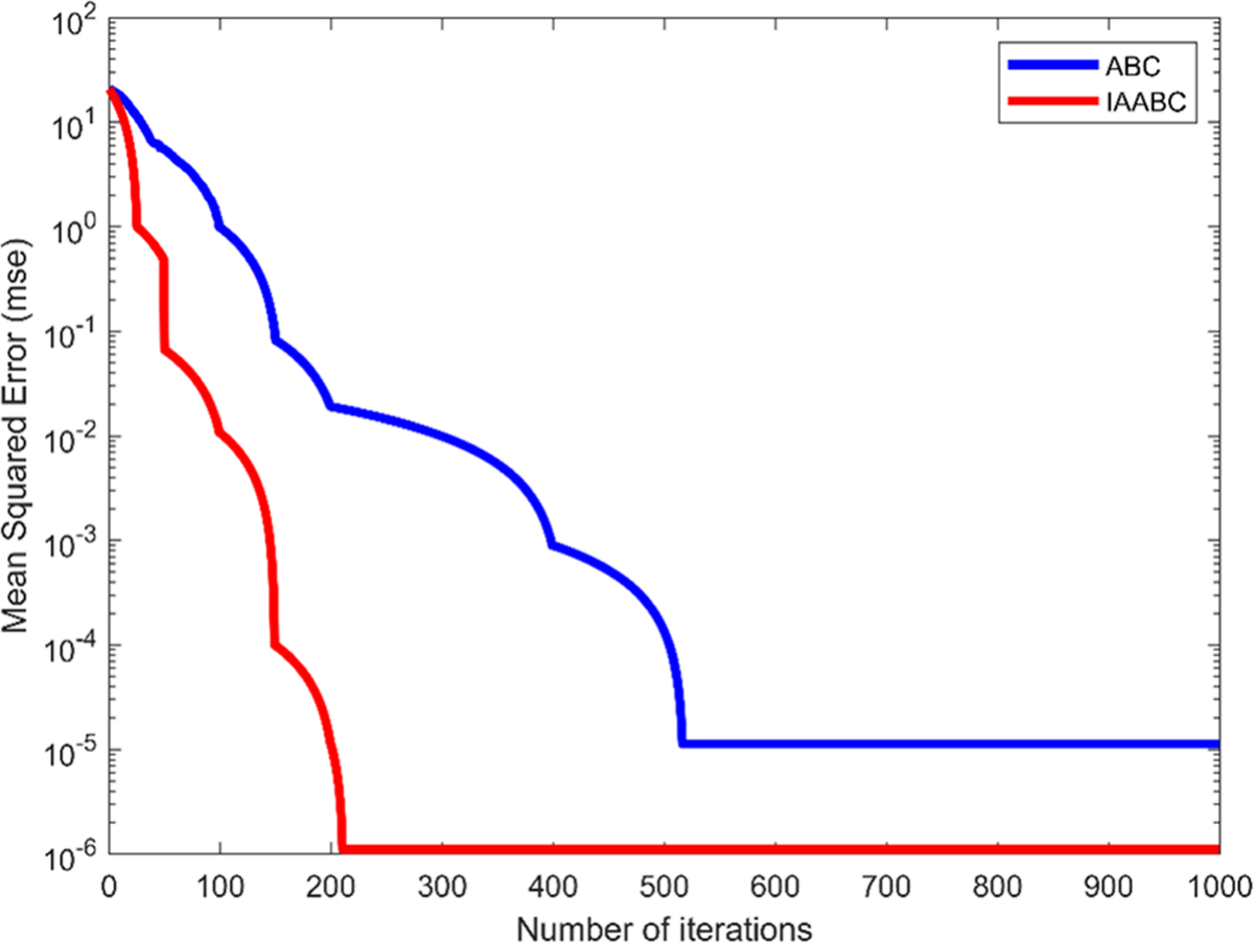
Comparison chart of IAABC and ABC optimization.

As can be seen from Figure 8, compared with the standard ABC algorithm, the IAABC algorithm can jump out of the local optimization when the standard ABC algorithm falls into the local optimization. The convergence speed and accuracy improve greatly and the IAABC algorithm has better stability after the adaptive factor and levy factor are added. The number of iterations of the IAABC algorithm approaching the target error value is significantly lower than that of the standard ABC algorithm.

To analyze and evaluate the classification performance of the IAABC-BP-ANN algorithm. DA, sigmoid SVM, RBF-SVM, BP-ANN, and ABC-BP-ANN were selected for comparison to classify the species of a single component. In addition, the score vectors of NaCl, NaOH, and PEA are used as the input variables, and the species of NaCl, NaOH, and PEA are used as the output variables. In particular, 1, 2, and 3 represent the output results of NaCl, NaOH, and PEA, respectively.

**Figure 9:**
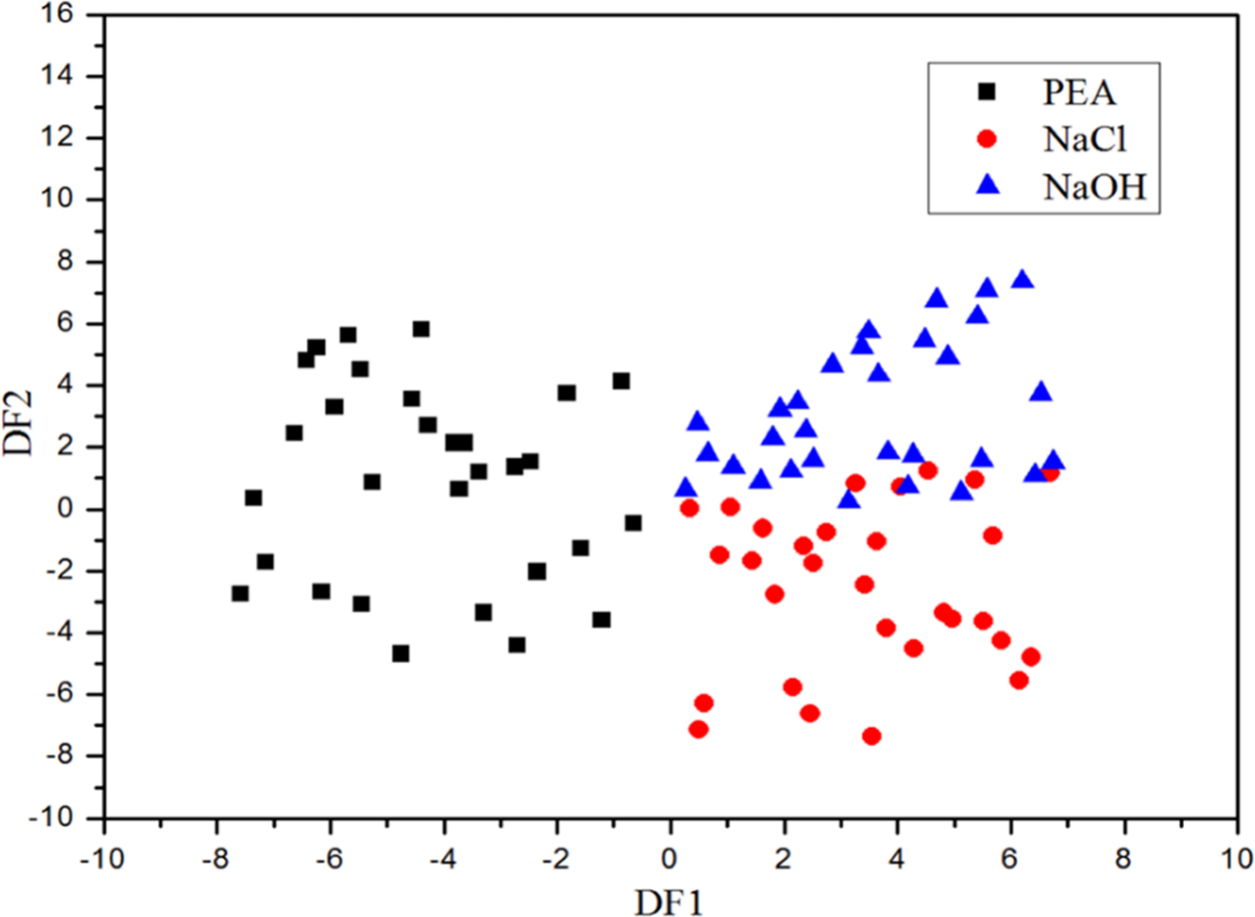
Sample distribution of NaCl, NaOH, and PEA in the plane of the discriminant functions.

DA is used to obtain two discriminant functions which are taken as the linear combination of input variables. Figure 9 shows the distribution of samples in the plane of the obtained discriminant function. As can be seen from Figure 9, the first discriminant function value of PEA is negative, and the first discriminant function values of NaCl and NaOH are both positive. Therefore, the species of NaCl, NaOH, and PEA can be distinguished according to the above results. However, the discriminant functions of NaCl and NaOH overlap partially, which can not distinguish them completely.

Table 1 shows the results that different performance indicators of NaCl, NaOH, and PEA for the testing set obtained by various classification models. As shown in Table 1, recall, precision, F-Score, and accuracy of the IAABC-BP-ANN algorithm are higher than other classification models. By using the IAABC-BP-ANN algorithm, recall, precision, F-Score and accuracy of NaCl, NaOH, and PEA are more than 98.5, 98.8, 0.986, and 97.9%, respectively. The result indicates that the IAABC-BP-ANN algorithm has better performance than other classification models.

**Table 1:** Different Performance Indicators of NaCl, NaOH, and PEA for the Testing Set Obtained by Various Classification Models^a^,^b^

	NaCl				NaOH				PEA			
model	*a*	*b*	*c*	*d*	*a*	*b*	*c*	*d*	*a*	*b*	*c*	*d*
1	82.5	78.6	0.805	84.3	83.7	86.8	0.852	82.9	85.3	89.2	0.872	88.7
2	87.4	82.6	0.849	86.3	86.2	88.4	0.873	85.1	88.3	90.6	0.894	90.2
3	88.1	82.9	0.854	86.6	86.3	88.6	0.874	85.4	88.3	90.8	0.895	90.4
4	90.7	89.4	0.900	89.2	90.6	90.2	0.904	89.6	91.4	92.3	0.918	92.3
5	94.8	95.3	0.950	95.6	94.7	95.7	0.952	95.2	95.4	95.8	0.956	96.1
6	98.7	98.9	0.988	97.9	98.5	98.8	0.986	98.2	99.1	99.3	0.992	98.9

a a,Recall (100%); b, Precision (100%); c, F-Score; d, Accuracy (100%).

b 1, DA; 2, Sigmoid SVM; 3, RBF-SVM; 4, BP-ANN; 5, ABC-BP-ANN; 6, IAABC-BP-ANN.

Meanwhile, the BP-ANN model is constructed, which is composed of three, eight, and three neurons in the input layer, hidden layer, and output layer, respectively. The transfer functions of the hidden layer and output layer are tansig and logsig, respectively, and the training function of back propagation is traingdm. As mentioned above, the IAABC algorithm sought the optimal threshold and weight by controlling the number of solutions (*N*), limit value (limit), and maximum number of iterations maxcycle (MaxCycle). It can be seen from the simulation results that the result is optimal when *N*, limit, and MaxCycle were 35, 100, and 2000, respectively.

According to the above results, BP-ANN, ABC-BP-ANN, and IAABC-BP-ANN are used to classify the species of their mixtures, respectively. In addition, the score vectors of their mixtures are used as the input variables, and the species of their mixtures are used as the output variables. In particular, 1, 2, 3, 4, 5, 6, and 7 represent the output results of 0, 10, 20, 30, 40, 50, and 60% m/m, respectively. Table 2 shows seven mixtures with different mole fractions (m/m).

**Table 2:** Seven Mixtures with Different Mole Fractions (m/m)

	mixture		
species (%)	NaCl (mol/L)	NaOH (mol/L)	PEA (mol/L)
0	0.0000124	0.0000392	0
10	0.0000154	0.0000409	0.00000625
20	0.0000267	0.0000107	0.00000936
30	0.0000324	0.0000077	0.0000172
40	0.0000445	0.0000047	0.0000328
50	0.0000315	0.000015	0.0000465
60	0.0000195	0.0000146	0.0000512

**Figure 10:**
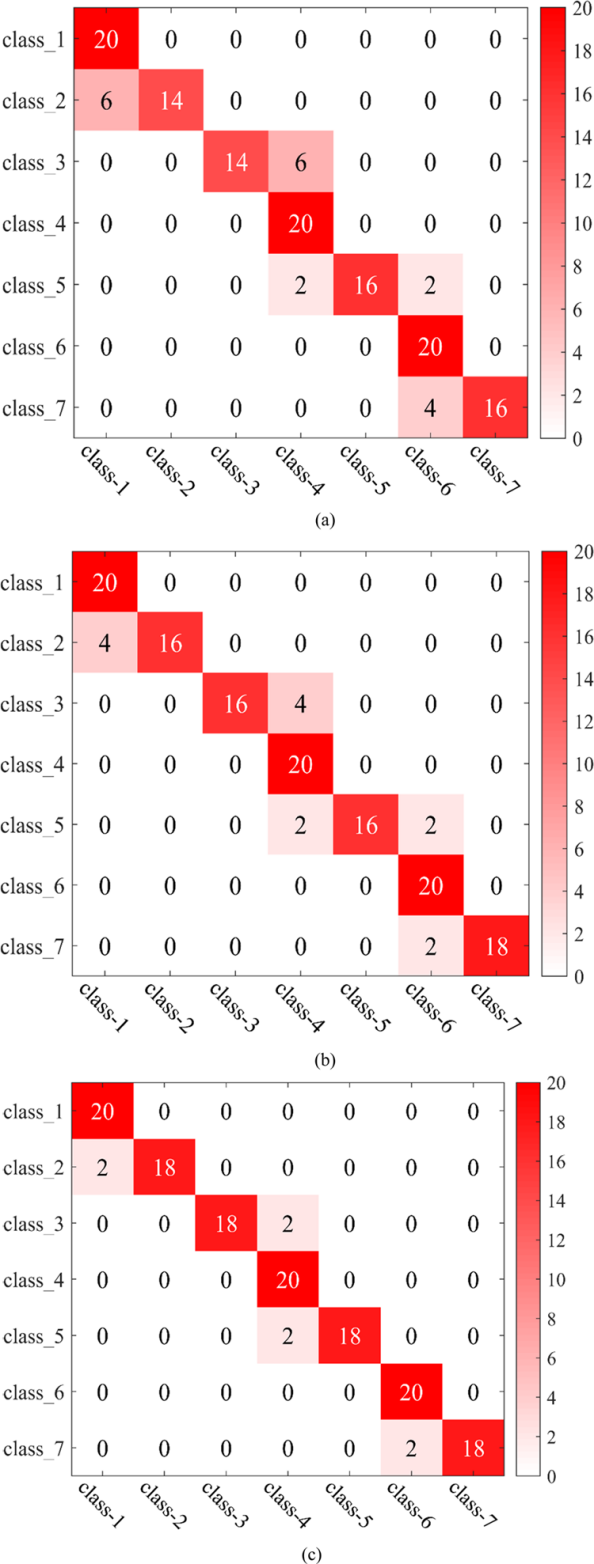
Classification results of the mixture obtained by three supervised classification methods: (a) BP-ANN; (b) ABC-BP-ANN; (c) IAABC-BP-ANN. Class-1, 0% m/m; class-2, 10% m/m; class-3, 20% m/m; class-4, 30% m/m; class-5, 40% m/m; class-6, 50% m/m; class-7, 60% m/m.

Figure 10 shows the classification results of the above three classification methods for testing samples of 140 groups for the mixture. As can be seen from Figure 10, misjudgment occurred at the four concentrations of 10, 20, 40, and 60, respectively. The main reason is that the score vectors of 10, 20, 40, and 60% m/m overlapped partially, and the boundaries of their score vectors are fuzzy (The categories of the boundary are mixed, and there is no obvious distinguishable boundary). Therefore, there are some misjudgments in the species prediction of 10, 20, 40, and 60% m/m.

Figure 10a shows the classification result obtained by the BP-ANN method. It can be seen that six samples of 10% m/m are misclassified as 0% m/m; six samples of 20% m/m are misclassified as 30% m/m; two samples of 40% m/m are misclassified as 30% m/m; another two samples of 40% m/m are misclassified as 50% m/m; and four samples of 60% m/m are misclassified as 50% m/m. Figure 10b shows the classification result obtained by the ABC-BP-ANN method. It can be seen that four samples of 10% m/m are misclassified as 0% m/m; four samples of 20% m/m are misclassified as 30% m/m; two samples of 40% m/m sample are misclassified as 30% m/m; another two samples of 40% m/m are misclassified as 50% m/m; and two samples of 60% m/m are misclassified as 50% m/m. Figure 10c shows the classification result obtained by the IAABC-BP-ANN method. It can be seen that two samples of 10% m/m are misclassified as 0% m/m; two samples of 20% m/m are misclassified as 30% m/m; two samples of 40% m/m are misclassified as 30% m/m; and two samples of 60% m/m are misclassified as 50% m/m.

From the above results, it can be concluded that the concentrations of the mixture samples which are misjudged are often relatively low. The main reason is that the absorption peaks of the mixture samples with low concentration are relatively weak or even disappeared. Compared with four evaluation indicators, recall, precision, F-Score, and accuracy, of the mixture in Table 3, it can be seen that the results obtained by the IAABC-BP-ANN method are the best, ABC-BP-ANN are the second, and BP-ANN are the worst. The results obtained from the mixture samples are consistent with those obtained from the single component.

**Table 3:** Different Performance Indicators of the Mixture for the Testing Set Obtained by Three Supervised Classification Models^a^

	recall			precision			F-score			accuracy		
species (%)	1	2	3	1	2	3	1	2	3	1	2	3
0	100	100	100	76.9	83.3	90.9	0.869	0.909	0.952	85.7	90.0	94.3
10	70	80	90	100	100	100	0.824	0.889	0.947	85.7	90.0	94.3
20	70	80	90	100	100	100	0.824	0.889	0.947	85.7	90.0	94.3
30	100	100	100	71.4	76.9	83.3	0.833	0.869	0.909	85.7	90.0	94.3
40	80	80	90	100	100	100	0.889	0.889	0.947	85.7	90.0	94.3
50	100	100	100	76.9	83.3	90.9	0.869	0.909	0.952	85.7	90.0	94.3
60	80	90	90	100	100	100	0.889	0.947	0.947	85.7	90.0	94.3

a 1, BP-ANN; 2, ABC-BP-ANN; 3, IAABC-BP-ANN.

Table 4 shows the results that the regression equation parameters of the mixture for the testing set obtained by three supervised classification models. As shown in Table 4, the RMSEP values of BP-ANN, ABC-BP-ANN, and IAABC-BP-ANN models are 0.1915, 0.1225, and 0.0816, respectively. The REP values obtained by BP-ANN, ABC-BP-ANN, and IAABC-BP-ANN models are 4.7871, 3.0619, and 2.0412, respectively. In addition, the correlation coefficients *R*^2^ obtained by BP-ANN, ABC-BP-ANN, and IAABC-BP-ANN models are 0.9781, 0.9864, and 0.9986, respectively. From the above results, it can be concluded that the RMSEP and REP values obtained by the IAABC-BP-ANN model are the smallest, and the corresponding error is the smallest. Meanwhile, the correlation coefficient *R*^2^ obtained by the IAABC-BP-ANN model is the largest, and the correlation is the strongest. Therefore, it can be seen that the IAABC-BP-ANN model has a better classification performance than BP-ANN and ABC-BP-ANN models.

**Table 4:** Regression Equation Parameters of the Mixture for the Testing Set Obtained by Three Supervised Classification Models

parameters	BP-ANN	ABC-BP-ANN	IAABC-BP-ANN
RMSEP	0.1915	0.1225	0.0816
REP (%)	4.7871	3.0619	2.0412
*R*^2^	0.9781	0.9864	0.9986

## 4 Conclusions

Based on the UV–vis spectra of NaCl, NaOH, PEA, and their mixtures, the absorbance values in the range 190–400 nm were used as input variables, and the supervised pattern recognition methods were used to identify the species of samples. First, PCA was used to extract the principal components of UV spectra for NaCl, NaOH, PEA, and their mixtures, and the obtained score vectors of the principal components were used as input variables. Then, several different supervised pattern recognition methods such as DA, sigmoid SVM, RBF-SVM, BP-ANN, ABC-BP-ANN, and IAABC-BP-ANN were compared. Finally, it can be known that the IAABC algorithm combined with BP-ANN obtained higher accuracy, recall, precision, and F-score than other classification methods. In addition, the RMSEP and REP values obtained by the IAABC-BP-ANN model are the smallest, and the correlation coefficient *R*^2^ obtained by the IAABC-BP-ANN model is the largest. The results proved the effectiveness of the proposed IAABC-BP-ANN method. Compared with other detection methods which used expensive equipment or involved the preparation of the cumbersome sample, UV spectrophotometry had some advantages such as easy availability, simple operation, low cost, easy maintenance, no secondary pollution, and so on. Therefore, it can be seen that UV spectroscopy combined with IAABC-BP-ANN is a simple, rapid, and reliable classification method for distinguishing NaCl, NaOH, PEA, and their mixtures. The research results provided a new theoretical basis and idea for the online synthesis and analysis of PEA.
